# Ferritin screening and Iron treatment for maternal anemia and fetal growth restriction prevention - A multicenter randomized controlled trial (FAIR Study)

**DOI:** 10.12669/pjms.39.1.6686

**Published:** 2023

**Authors:** Tayyiba Wasim, Natasha Bushra, Arif Tajammul, Shamsa Humayun, Saba Rasool, Fatima Shahbaz, Anam Riaz, Farah Siddique, Khadija Irfan Khawaja, Aziz Fatima, Zubia Zafar, Khalid Saeed Khan

**Affiliations:** 1 Tayyiba Wasim, MBBS, FCPS,. Department of Obstetrics & Gynaecology Services, Institute of Medical Sciences, Services Hospital, Lahore, Pakistan; 2 Natasha Bushra, MBBS, FCPS, Department of Obstetrics & Gynaecology Services, Institute of Medical Sciences, Services Hospital, Lahore, Pakistan; 3 Arif Tajammul, MBBS, MRCOG., Department of Obstetrics & Gynecology, Allama Iqbal Medical College, Jinnah hospital, Lahore, Pakistan; 4 Shamsa Humayun. MBBS, FCPS, Department of Obstetrics & Gynecology, Fatima Jinnah medical College, Ganga Ram Hospital, Lahore, Pakistan; 5 Saba Rasool, MBBS, FCPS, Department of Obstetrics & Gynaecology Services, Institute of Medical Sciences, Services Hospital, Lahore, Pakistan; 6 Fatima Shahbaz, MBBS, FCPS, Department of Obstetrics & Gynecology, Fatima Jinnah medical College, Ganga Ram Hospital, Lahore, Pakistan; 7 Anam Riaz, MBBS , Department of Obstetrics & Gynaecology Services, Institute of Medical Sciences, Services Hospital, Lahore, Pakistan; 8 Farah Siddique, MBBS, FCPS, Department of Obstetrics & Gynecology, Allama Iqbal Medical College, Jinnah hospital, Lahore, Pakistan; 9 Khadija Irfan Khawaja, MBBS, FCPS, Department of Endocrinology Services Institute of Medical Sciences, Services Hospital, Lahore, Pakistan; 10 Aziz Fatima, MBBS, FCPS, Department of Endocrinology Services Institute of Medical Sciences, Services Hospital, Lahore, Pakistan; 11 Zubia Zafar Department of Endocrinology Services Institute of Medical Sciences, Services Hospital, Lahore, Pakistan; 12 Khalid Saeed Khan, MBBS, MRCOG. Department of Preventive Medicine and Public Health, Faculty of Medicine, University of Granada, Granada, Spain

**Keywords:** Non anemic iron deficiency, Maternal anemia, Fetal weight restriction

## Abstract

**Background:**

Non-anemic iron deficiency precedes iron deficiency anaemia and has an estimated prevalence of 1-2 billion worldwide. Few studies have comprehensively researched the idea of treating non-anemic iron deficiency (NAID) with iron to improve the outcome of the mother and the offspring.

**Methods and Analysis:**

FAIR will be a multicenter randomized controlled trial that will be conducted in multiple clinical academic obstetrics units in Lahore (including Services Institute of Medical Sciences, Lahore, Allama Iqbal Medical College, Lahore and Fatima Jinnah Medical University). Pregnant women at gestational age <20 weeks with hemoglobin 11-13 g/L and ferritin below the threshold (<30 ng/ml) will be invited to take part in the study. Randomization will be done by computer based generated random numbers. One group (usual care or oral group) will be offered routine care prophylactic dose of oral iron (30-45 mg/day) and the other group (intervention arm or IV group) will be offered therapeutic dose of IV iron (dose calculated by Ganzoni formula) in addition to usual care. All patients will be followed up till delivery. Primary maternal outcome will be hemoglobin at 36 weeks’ gestation. Secondary outcomes are fetal birthweight or small for gestational age, preterm birth, preeclampsia, multidimensional fatigue inventory, breast feeding initiation, blood transfusion, and fetal cord ferritin and hemoglobin.

**Discussion:**

The study will generate evidence as to whether screening serum ferritin in non-anemic pregnant women and replenishing their iron stores will likely reduce the rate of predelivery anemia in pregnant women, improve birthweight and preventing perinatal complications.

**Roles and responsibilities:**

Tayyiba Wasim is principal Investigator and other members of data management team are Natasha Bushra, Shamsa Humayoun, Khalid Saeed Khan, Fatima Shehbaz, Saba Rasool, Anam Riaz and Sonia Irshad. Principal investigator will assume the full responsibility of Fair trial including training of research assistants, administration of informed consent and protecting participants confidentiality. Data management team will help in the management, development and execution of trial. Khadija Irfan Khawaja is the operational lead for fair trial´s technology team comprising of Aziz Fatima and Zubia Zafar, responsible for gathering requirements from study teams and supporting the operational implementation of technology to drive the collection of high-quality study data. Protocol contributors are Gynae unit I of Services Institute of Medical Sciences/ Services hospital, Lahore, Gynae Unit II of Allama Iqbal Medical College/ Jinnah hospital, Lahore and Gynae unit 1 of Fatima Jinnah Medical College/ Sir Ganga Ram hospital, Lahore. These coordinating centres will recruit patients (sample size=600) and will discuss their progress in trial management meetings quarterly.

**Steering committee:**

has an independent chair Prof Samia Malik, one expert member Prof Faiza Bashir and Ms Neelam to represent patients, public and consumers. Trial steering committee with independent chair and member with a patient representative will oversee the study. Chair of steering committee has the authority to stop the trial whenever needed in case of positive or negative results. Steering committee meetings will be held on annual basis.

**Independent Data monitoring committee:**

comprises of Dr. Shehnoor Azhar as chair and Prof Ejaz Hussain and Dr. Shehla Javed Akram as members. Data monitoring committee will assess the progress, data safety and if needed critical efficacy points of the clinical study and will show their results quarterly in data interim meetings. The committee will focus on integrity of the whole process and compliance of all sites with all aspects of the protocol. It will perform confidential interim analyses quarterly, which may be used to determine if an effect is observed and if the study should continue to its planned sample size. Data monitoring committee will report to the Chair of the steering committee.

**Trial Registration No:** NCT04228627 www.clinicaltrials.gov (Dated 14 January 2020)

**Version Date/ No:** Version 1.3 Updated 06.05.2021

## INTRODUCTION

Anemia is the world’s second leading cause of disability with a global prevalence of 38% amongst pregnant women.^1^ It affects more than 56 million women worldwide, two-thirds being from Asia.^2^ Iron deficiency accounts for majority of the cases and is the most common cause of anemia worldwide. Iron requirements increase 10-fold during pregnancy from 6 mg/day to 22 mg/day. Diet alone cannot meet this huge demand, so first the iron stores are used up and then iron deficiency aneemia sets in. Studies from developed countries also report prevalence of iron deficiency among 24-40% of women.^3^,^4^ This situation is escalated in low-middle income countries (LMIC) where women enter pregnancy with low stores, hence they develop iron deficiency anemia early and more severely. The prevalence of anemia in pregnant women in LMIC is estimated to be 45-50%.^5^-^7^ Multiple factors including iron deficient diet, short inter pregnancy interval and parasitic infections are responsible.

Iron deficiency anemia is detrimental to pregnant mothers both in terms of physical and mental wellbeing. It is associated with antepartum, intrapartum and postpartum morbidity in terms of increased fatigue, vertigo, leg cramps, irritability, postpartum hemorrhage, increased chances of sepsis and postpartum depression.^8^,^9^ The fetus is entirely dependent on maternal iron stores or daily maternal iron absorption for its supply of micronutrients. Offspring compromise in terms of low birth weight, prematurity, growth restriction, increased perinatal mortality and neonatal anemia subsequent to birth is reported.^10^,^11^ Children born to anemic mothers are reported to have low IQ at seven years of age.^12^ Birth weight is an important determinant of child survival, and low birth weight children are at increased risk of ill health and mortality.

WHO defines anemia in pregnancy as hemoglobin < 11gm/dl in any trimester.^13^ UK antenatal guidelines define hemoglobin less than 11 gm/dl in first trimester and <10.5 gm/dl in second and third trimester as anemia in pregnancy.^14^ Iron deficiency is tested by a laboratory test of ferritin and complete blood count (CBC) including hemoglobin concentration and red blood cell indices. International Federation of Gynecology and Obstetrics (FIGO) recommends a minimum prophylactic dose of 30 mg/day iron supplementation in pregnancy in areas of high prevalence of anemia and 100-200mg /day is recommended as treatment in mothers with established iron deficiency anemia.^15^ Pregnancies with iron deficiency anemia are treated according to established protocols with oral and parenteral iron.^15^-^18^ Oral iron is substituted with parenteral iron in women who do not tolerate oral preparations well. Daily prenatal use of iron supplementation has been reported to result in improved birth weight in a linear dose response fashion.^19^ World Health Assembly has set a target of 50% reduction of anemia by 2025. A reference document has been designed by WHO for policy makers to implement programs of detection and control of anemia at regional and national levels.^20^

There is a spectrum of iron deficiency in pregnancy ranging from iron deficiency without anemia to iron deficiency anemia. Non-anemic iron deficiency (NAID) precedes iron deficiency anaemia and has estimated prevalence of 1-2 billion worldwide.^21^ All pregnant women at their booking visit are offered CBC but routine testing of serum ferritin is not common in clinical practice. Serum Ferritin is the most sensitive test of iron deficiency and low levels reflect a state of depleted iron stores.^22^ Different thresholds of serum Ferritin are described for diagnosis of iron deficiency anemia in various studies.^23^ Recent UK guidelines on management of anemia in pregnancy recommend that non anemic women at risk of iron deficiency should be identified and have their serum ferritin checked. Serum Ferritin < 30L is indicative of iron deficiency.^24^ The test for iron deficiency with serum ferritin level <30 /L has sensitivity of 92% and specificity of 98%. Few studies have comprehensively researched the idea of treating NAID with iron to improve the outcome of mother and offspring. Studies on NAID suggests a variety of possible benefits of iron supplementation on infant birth, educational attainment, infant development and maternal fatigue.^25^ There is lack of robust evidence that treatment of NAID will result in significant benefit to mother and fetus in terms of prevention of low birth weight. The clinical consequences of NAID in pregnancy are largely unknown and there is a need to determine through research if there is a need for a national screening program for early detection and intervention.^26^ There is multidisciplinary willingness to put this hypothesis to test in a robust trial.^27^ All woman who book themselves at antenatal hospitals if screened with a serum ferritin test can be offered therapeutic doses of iron, which can lead to prevention of maternal and fetal complications of iron deficiency.

The rationale of this study is to observe the outcome of NAID in pregnant woman in terms of hemoglobin at 36 weeks’ gestation in addition to secondary outcomes including birth weight, preterm birth, development of preeclampsia in the mother, multidimensional fatigue inventory (MFI), breast feeding initiation, blood transfusion, fetal cord blood ferritin and hemoglobin. It is aimed at the hypothesis that early screening and treatment of iron-depleted non-anemic women will reduce the risk of developing maternal anemia and prevent complications like low birth weight or small according to their gestational age in babies. This is the first trial of its kind in Pakistan.

### Hypothesis:

The NAID women who are given therapeutic doses of IV iron will more often have normal maternal haemoglobin at term and normal birth weight of the baby as compared to the NAID women who are given usual care.

### Trial design:

It is a parallel, two-arm (intervention-control) and double blind (care providers and outcome assessors both blind) randomised controlled trial of IV Iron intervention provided to NAID women to replenish their iron stores compared with usual care with prophylactic oral iron. Eligible participants are randomized to the intervention and usual care groups. A 600 sample size will be recruited from three centers. All patients will be followed up till delivery. Post randomisation evaluation will be done at 28 and 36 weeks regarding maternal hemoglobin, serum ferritin, MFI scoring, obstetrical scan for fetal parameters, blood transfusion, development of any complication (preeclampsia, preterm labor, IUGR) and fetal birth weight. Fetal cord hemoglobin and serum ferritin will be checked after delivery. In this way, assessment for primary and secondary outcomes will be done and data set prepared for analysis.

## METHODS

### Participants, Intervention and outcome:

### Study setting:

FAIR trial will be conducted in respective departments of obstetrics of three teaching hospitals of Lahore including Services Institute of Medical Sciences and its Services Hospital, Allama Iqbal Medical College and its Jinnah Hospital, and Fatima Jinnah Medical University and its Sir Ganga Ram Hospital.

### Eligibility Criteria:

Selection criteria is established using the PICO or population, intervention, comparison and outcome framework explained in Table-I.

**Table-I:** PICO for study population.

	Population	Intervention	Comparison	Outcome
Inclusion	Singleton pregnancy Haemoglobin 11-13 g/L at booking with ferritin below the threshold (<30) Age >18 years of age at recruitment to the study Able to provide written informed consent Patients willing to enroll in study	NAID patients will be divided into two groups by randomization. One group (usual care/oral group) will be offered the routine care prophylactic dose of oral iron which is 30-45 mg and other group (interventional /IV group) will be offered intravenous therapeutic dose of iron to replenish iron stores which is 1000mg calculated by Ganzoni’s formula. They will continue the prophylactic dose after replenishing iron stores. Adherence to intervention protocols will be monitored by proper documentation and laboratory test	Usual care vs intervention arm will be compared for outcome	Primary: Maternal: Hemoglobin at 36 weeks’ gestation to birth Secondary: Neonatal birthweight or small for gestational age, preterm birth, blood transfusion, preeclampsia, multidimensional fatigue inventory, breast feeding avoidance/initiation, neonatal cord ferritin and haemoglobin

### Participant Timeline:

**Fig.1:**
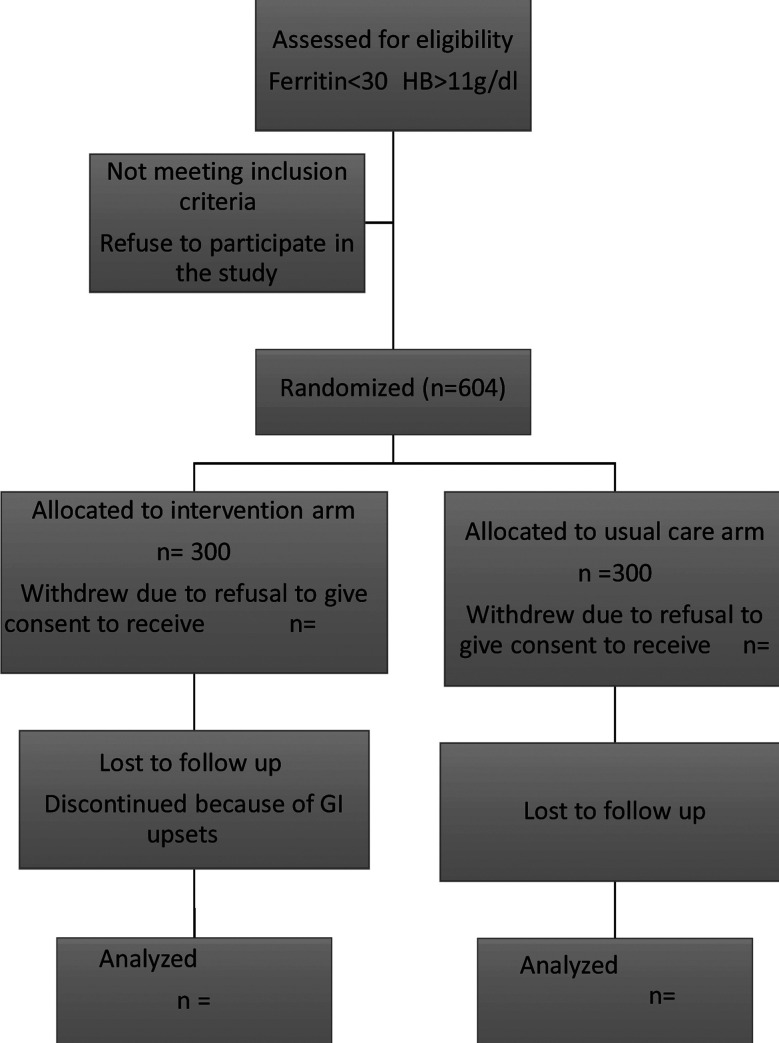
Flow of participants throughout the FAIR study.

From booking (<20 weeks) till delivery of the fetus (Fig.1).

### Sample Size:

Using haemoglobin at 36 weeks as a continuous outcome measure and to confirm or refute a small to medium effect of screening/intervention vs usual care (0.3 SD difference), based on ? = 0.05 and β = 0.1 (90% power), 235 patients in each group (i.e. 470 patients in total) will be required. Considering a 20% loss to follow-up, the sample size is inflated to 300 patients in each group (i.e. 600 patients in total).

### Recruitment:

Patient recruitment service providers in outpatient departments will educate the public about the value of clinical trial participation and will encourage the enrollment. All singleton pregnant women with gestational age <20 weeks coming to outdoor department of respective hospitals will be screened for hemoglobin and serum ferritin. If their hemoglobin ≥11g/dl and serum ferritin concentration <30ug/l they will be enrolled for trial after taking informed consent and randomization will be carried out.

### Methods: Assignment of Interventions

### Allocation:

After recruiting the participants, their data will be entered by investigators sitting in outpatient department in a online computer software program made by technology team that will assign a pin code to every participant which will help in follow up entry of each participant´s data and data analysis by technology team. After randomization, computer software will automatically generate numbers to the participants in an allocation ratio of 1:1 to each group, it will help remove the selection bias. Numbers will be generated from 1-25 randomly by software and after completion of first 25 participants the program will automatically start a new set of 25 numbers. Concealed boxes in sets of 1-25 having IV and oral iron will be made by another team of investigators in respective wards which will be given to the participant having the same computer software generated number.

### Blinding:

Researchers and care givers will be blinded about the allocation of each participant either to the intervention or usual care group. Unblinding will be permissible to care givers at 28 weeks for further entry of data of allocated participant in respective groups.

### Data Collection and Management:

All pregnant women of gestational age <20 weeks in the participating units will be advised complete blood count. Those having haemoglobin >11g/dl, will have their serum ferritin levels checked. Pregnant women having serum ferritin <30μg/l will be counselled for enrolment into the study and informed consent will be taken after explaining the details of the study. They will be divided into two groups randomly. Therapeutic dose of IV iron will be advised to intervention group and prophylactic dose of oral iron (30-45 mg) will be given to the control group of pregnant women to be continued till delivery. All pregnant women will be followed up till delivery with the usual protocol of monthly visits till 28 weeks then fortnightly visits till 36 weeks and then weekly. Haemoglobin and serum ferritin in both arms will be measured at 28 weeks and 36 weeks of gestation. Development of preeclampsia, preterm birth, blood transfusion, fatigue score, birth weight along with cord blood haemoglobin and serum ferritin will be recorded. Data will be entered into an electronic database (computer software program ASP. NET MVC architecture data collection platform based on SQL 2014 for data normalization) has been purpose designed for the trial with defined multi level user access and separate case report forms for each study visit. The software platform ensures transparent randomisation, allows real-time data acquisition and smooth tracking of the enrolled participants to facilitate standardised, and synchronous data collection across all the study sites, enabling data quality checks and ensuring authenticity of data.

### Data Analysis:

All the statistical analysis will be done on statistical package for social sciences (SPSS). Mean and standard deviation will be calculated for continuous variables like age, gestational age, haemoglobin, serum ferritin levels etc. Frequency and percentages will be calculated for categorical variables like gender. If data are not normally distributed then median will be used. Confounders like age, duration of disease and co morbidities will be controlled through multivariable analysis documented through a statistical analysis plan prepared before the outcome data per group are unblinded. Comparison of outcome will be tested by chi-square and student’s t-test. P value of <0.05 will be significant.

### Data Monitoring:

Trial steering committee with an independent chair and membership including a patient representative will oversee the study. Data monitoring committee will perform confidential interim analyses, which may be used to determine if an effect is observed and if the study should continue to its planned sample size. Any adverse reaction to IV Iron or blood transfusion will be noted and entered in specific transfusion reaction registers in wards according to hospital protocol. Independent data monitoring committee will systematically review all trial related activities, data entry, will assess compliance with regulatory requirements for clinical trial protocol and clinical quality management plan by principal investigator and all sites support personnels and audit trial quarterly to identify potential risks and help to ensure that compliance issues do not jeopardize the trial.

### Patient and public involvement:

A lay patient representative (patient with lived experience) has been included in the trial steering committee. The representative has given advice on the protocol and consent procedures submitted for ethics committee approval and will give oversight to the study during its conduct.

### Ethics and Dissemination:

The study will tell us whether by screening serum ferritin early in nonanemic pregnant women and replenishing their iron stores will reduce the incidence of anemia in pregnant women, improve birthweight and preventing complications.

### Ethics Approval:

Ethics approval has been granted by institutional review board of Services Institute of Medical Science, Lahore: IRB/2019/599/SIMS (dated 05 December 2019), Allama Iqbal Medical College, Lahore: 54^th^/ERB (dated 17 December 2019) and Fatima Jinnah Medical University, Lahore; 34-Res-Proposal-Gynae-1/ERC (dated 29 October 2020). Appendix No.1.

### Consent:

Informed consent will be taken by the investigators conducting the trial in outpatient departments on specially designed consent proformas. Appendix No.2

### Declaration of Interest:

None.

### Access to Data:

Data monitoring team, Trial Technology team will be given access to the data.

### Dissemination Policy:

Trial findings will be published in peer reviewed journals and propagated in different conference meetings, providing much needed evidence to inform clinical and public health actions in this area. Dissemination will also be done through amendments in ward protocols.

Protocol prepared according to Spirit checklist (https://www.spirit-statement.org/)^28^
